# *Aedes aegypti* Piwi4 Is a Noncanonical PIWI Protein Involved in Antiviral Responses

**DOI:** 10.1128/mSphere.00144-17

**Published:** 2017-05-03

**Authors:** Margus Varjak, Kevin Maringer, Mick Watson, Vattipally B. Sreenu, Anthony C. Fredericks, Emilie Pondeville, Claire L. Donald, Jelle Sterk, Joy Kean, Marie Vazeille, Anna-Bella Failloux, Alain Kohl, Esther Schnettler

**Affiliations:** a MRC-University of Glasgow Centre for Virus Research, Glasgow, Scotland; b Department of Microbiology, Icahn School of Medicine at Mount Sinai, New York, New York, USA; c Roslin Institute, University of Edinburgh, Edinburgh, Scotland; d Arboviruses and Insect Vectors Unit, Department of Virology, Institut Pasteur, Paris, France; Boston University School of Medicine

**Keywords:** *Aedes aegypti*, PIWI, RNA interference, antiviral response, arbovirus, innate immunity

## Abstract

The small interfering RNA (siRNA) pathway is a major antiviral response in mosquitoes; however, another RNA interference pathway, the PIWI-interacting RNA (piRNA) pathway, has been suggested to be antiviral in mosquitoes. Piwi4 has been reported to be a key mediator of this response in mosquitoes, but it is not involved in the production of virus-specific piRNAs. Here, we show that Piwi4 associates with members of the antiviral exogenous siRNA pathway (Ago2 and Dcr2), as well as with proteins of the piRNA pathway (Ago3, Piwi5, and Piwi6) in an *Aedes aegypti*-derived cell line, Aag2. Analysis of small RNAs captured by Piwi4 revealed that it is predominantly associated with virus-specific siRNAs in Semliki Forest virus-infected cells and, to a lesser extent, with viral piRNAs. By using a Dcr2 knockout cell line, we showed directly that Ago2 lost its antiviral activity, as it was no longer bound to siRNAs, but Piwi4 retained its antiviral activity in the absence of the siRNA pathway. These results demonstrate a complex interaction between the siRNA and piRNA pathways in *A. aegypti* and identify Piwi4 as a noncanonical PIWI protein that interacts with members of the siRNA and piRNA pathways, and its antiviral activities may be independent of either pathway.

**IMPORTANCE** Mosquitoes transmit several pathogenic viruses, for example, the chikungunya and Zika viruses. In mosquito cells, virus replication intermediates in the form of double-stranded RNA are cleaved by Dcr2 into 21-nucleotide-long siRNAs, which in turn are used by Ago2 to target the virus genome. A different class of virus-derived small RNAs, PIWI-interacting RNAs (piRNAs), have also been found in infected insect cells. These piRNAs are longer and are produced in a Dcr2-independent manner. The only known antiviral protein in the PIWI family is Piwi4, which is not involved in piRNA production. It is associated with key proteins of the siRNA and piRNA pathways, although its antiviral function is independent of their actions.

## Abstract (precis)

Mosquitoes transmit several pathogenic viruses, for example, the chikungunya and Zika viruses. In mosquito cells, virus replication intermediates in the form of double-stranded RNA are cleaved by Dcr2 into 21-nucleotide-long siRNAs, which in turn are used by Ago2 to target the virus genome. A different class of virus-derived small RNAs, PIWI-interacting RNAs (piRNAs), have also been found in infected insect cells. These piRNAs are longer and are produced in a Dcr2-independent manner. The only known antiviral protein in the PIWI family is Piwi4, which is not involved in piRNA production. It is associated with key proteins of the siRNA and piRNA pathways, although its antiviral function is independent of their actions.

## INTRODUCTION

Viruses transmitted by arthropod vectors to vertebrate hosts are known as arboviruses, and they are spread by vectors, including mosquitoes, ticks, midges, and sandflies. Arboviruses most commonly belong to the *Bunyaviridae*, *Togaviridae*, *Flaviviridae*, and *Reoviridae* families, and in many instances they greatly impact human and animal health. Mosquitoes of the *Aedes* genus transmit the human-pathogenic chikungunya (CHIKV), dengue (DENV), and Zika (ZIKV) viruses. Arboviruses actively replicate not only in the vertebrate host but also in infected vectors, which in turn also mount antiviral immune responses (1). The major antiviral response in mosquitoes is a sequence-specific RNA breakdown mechanism called RNA interference (RNAi). It can be divided into several pathways that differ in the nature of their induction, effector proteins, and small RNA molecules: small interfering RNA (siRNA), microRNA (miRNA), and PIWI-interacting RNA (piRNA). The exogenous siRNA (exo-siRNA) pathway is considered to be the main antiviral response. It is triggered through dicer 2 (Dcr2) recognition of virus double-stranded RNA (dsRNA), which is processed into 21-nucleotide (nt)-long virus-specific siRNAs (vsiRNA) that are unwound and loaded into Argonaute 2 (Ago2) in the multiprotein RNA-induced silencing complex (RISC). It is assumed that the complementary strand of the vsiRNA duplex is degraded, while the remaining strand guides Ago2 to complementary viral RNA, followed by cleavage and degradation of the target RNA. Arbovirus-specific vsiRNAs have been reported for a variety of arbovirus-infected mosquitoes, and Ago2 in particular has been shown to play a key role in the antiviral response, as its knockdown enhances virus replication (2–7).

Recently, piRNA-like molecules (25 to 30 nt in length) that mapped to virus genome sequences were found to be produced in infected *Drosophila melanogaster* cells (8). The piRNA pathway has since been implicated in antiviral responses in mosquitoes, and virus-specific piRNA molecules have been identified in arbovirus-infected mosquitoes and their derived cells (9–15). Most of our knowledge of the insect piRNA pathway comes from studies conducted with *D. melanogaster*, in which primary piRNAs are synthesized initially in the form of long transcripts from genomic regions (16, 17) and, upon their cleavage, primary piRNAs (with a uridine at position 1 [U_1_], bound to Aub or Piwi) are produced and target transposon RNAs. This cleavage results in the production of secondary piRNAs (with an adenine at position 10 [A_10_]), which bind to Ago3. In turn, Ago3 targets antisense RNA transcripts, which results in the production of primary-type secondary piRNAs. This production is called the ping-pong mechanism of piRNA production (18–21). Ago3, Aub, and Piwi themselves are members of the PIWI family within the AGO clade (22).

Compared to *D. melanogaster*, the piRNA pathway in *A. aegypti* shows notable differences. First, in the fly model, piRNAs are predominantly produced in germline cells due to the restricted expression of PIWI proteins Piwi, Aub, and Ago3 (23, 24) and play an important role in silencing transposons and maintaining genome stability. In comparison, in aedine mosquitoes, piRNAs are present in both germline and somatic tissues (12). Furthermore, *A. aegypti* lacks the Aub gene, but the genome encodes 7 different PIWI proteins (Piwi1 to -7) (13, 25). Virus-derived piRNA-like small RNAs (vpiRNAs) show the characteristic ping-pong motif and have been found in *Aedes*-derived cells as well as in somatic tissues in mosquitoes (6, 10–12, 15). Although no antiviral activity could be linked to the piRNA pathway in *D. melanogaster* (26), knockdown of Piwi4 in the *A. aegypti*-derived cell line Aag2 resulted in increased replication of the model mosquito-borne arbovirus of the *Togaviridae* family, the alphavirus Semliki Forest virus (SFV) (13). Intriguingly, Piwi4 was not needed for the production of vpiRNAs specific for SFV, Sindbis virus (SINV), or DENV infections (13, 27, 28). Indeed, the importance of Ago3, Piwi5, and Piwi6 in the production of SINV- and DENV-specific vpiRNAs, as well as in the binding of genomic vpiRNAs with an A at position 10 by Ago3 and antigenomic vpiRNAs with a U at position 1 by Piwi5 and Piwi6, has been demonstrated. Significantly, it was reported that Piwi4 does not bind piRNAs (27, 28). In a different study it was found that silencing of these proteins (Piwi5, Piwi6, and Ago3) had only minor effects on SFV replication (13). All these studies highlighted that the antiviral role of the piRNA pathway in *A. aegypti* remains poorly understood; in particular, the role(s) or activity of Piwi4 in these antiviral responses remains enigmatic.

Here, we analyzed the properties of Piwi4 in more detail. By studying SFV infection of *A. aegypti*-derived Aag2 cells and performing pulldown experiments, in combination with small RNA sequencing, we further elucidated Piwi4 functions. We found that Piwi4 was associated with Ago3, Piwi5, and Piwi6 proteins of the piRNA pathway, in addition to Ago2 and Dcr2 of the exo-siRNA pathway. Piwi4 is predominantly associated with vsiRNAs, although the use of Dcr2 knockout (KO) cells suggested that its antiviral activity was independent of Dcr2 and thus also the exo-siRNA pathway. This suggests a potentially novel function of Piwi4 and antiviral activity outside canonical RNAi pathways.

## RESULTS

### Piwi4 associates with proteins of the siRNA and piRNA pathways.

**FIG 1:**
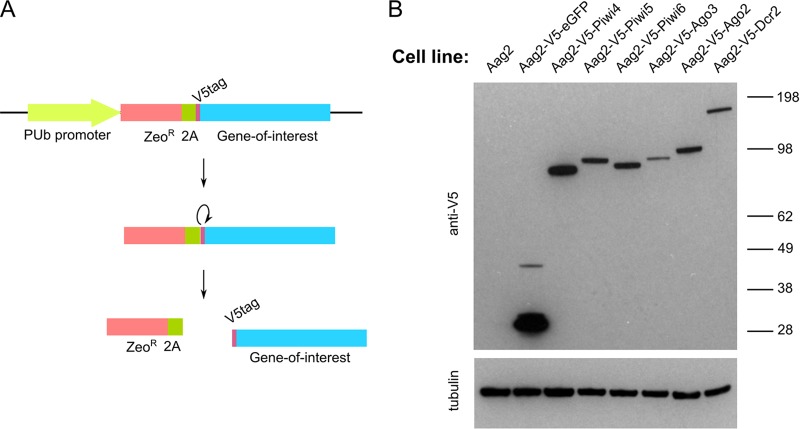
Stable expression of tagged RNAi proteins in *A. aegypti-*derived Aag2 cells. (A) Schematic representation of constructs used for production of stable cell lines. Polycistronic mRNA was expressed from the polyubiquitin promoter (PUb), and zeocin (Zeo) was cleaved from the gene of interest via 2A autoprotease activity of *Thosea asigna* virus. Linearized plasmid was transfected into Aag2 cells, followed by zeocin selection. (B) Immunoblot analysis of cell line extracts for the expression of V5-tagged siRNA and piRNA pathway proteins and eGFP (control). Tubulin was used as a loading control.

Due to the lack of antibodies against *A. aegypti* PIWI proteins, Aag2 cell lines stably expressing V5-tagged PIWI proteins were produced. For this, an expression cassette encoding the zeocin resistance marker and tagged protein divided by two 2 A autoprotease sequences (derived from insect-specific *Thosea asigna* virus [29]) was cloned into the pPUb plasmid behind the *A. aegypti* polyubiquitin (PUb) promoter (30) (Fig. 1A). Using this cassette, stable Aag2 cell lines were produced that expressed V5-tagged Piwi4, Piwi5, Piwi6, Ago3, Ago2, Dcr2, and enhanced green fluorescent protein (eGFP, as a control) (Fig. 1B).

**FIG 2:**
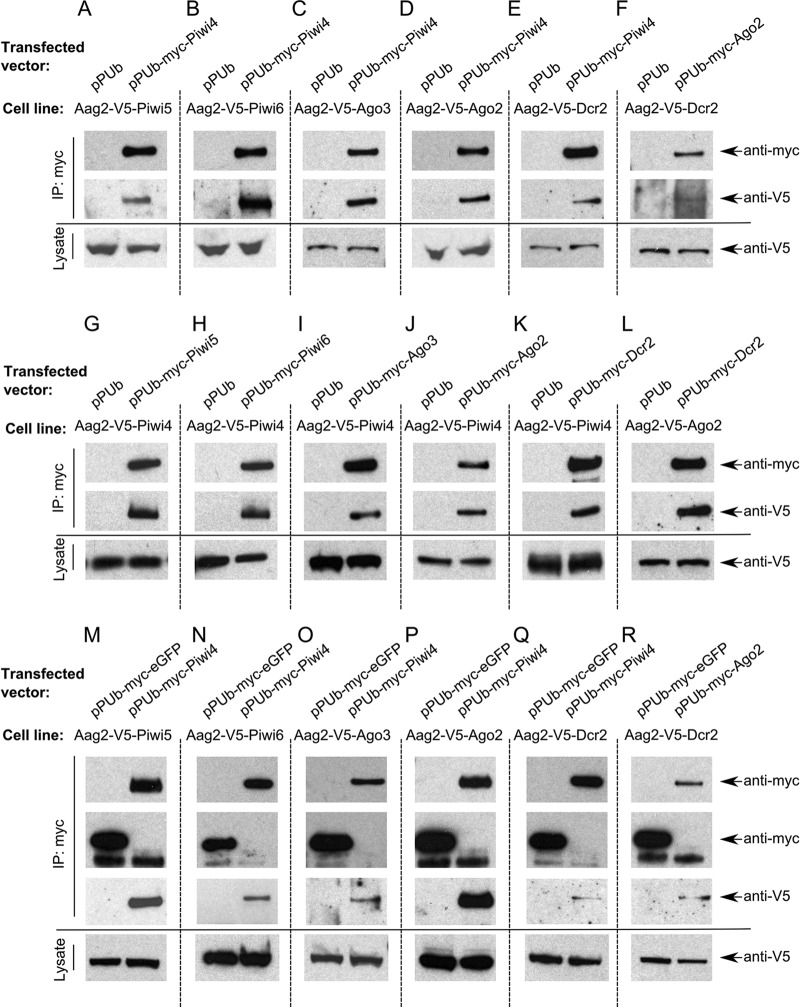
Analysis of RNAi protein associations in Aag2 cells. V5-tagged protein-expressing Aag2 cell lines were mock transfected (pPUb) or transfected with a myc-tagged protein expression construct; cell lysates were prepared 48 h p.t. and subjected to immunoprecipitation (IP) with anti-myc antibodies. Samples were analyzed by Western blotting. Immunoprecipitated samples were probed for the presence of V5 and myc tags, and cell lysates were analyzed for V5 tag. Associations between Piwi4 and Piwi5 (A, G, and M), Piwi6 (B, H, and N), Ago3 (C, I, and O), Ago2 (D, J, and P), or Dcr2 (E, K, and Q) and between Ago2 and Dcr2 (F, L, and R) are shown.

To determine if Piwi4 interacts with any of these V5-tagged RNAi proteins, stable cell lines were transfected with an expression construct encoding myc-tagged Piwi4, followed by pulldown assays with myc-Piwi4 and Western blotting using myc and V5 tag-specific antibodies. All PIWI proteins investigated (Piwi5, Piwi6, and Ago3 [Fig. 2A, B, and C]) could be detected in the pulldown products with myc-tagged Piwi4. These results were confirmed in reciprocal experiments, e.g., based on pulldown of myc-tagged Piwi5 and detection of V5-tagged Piwi4 (Fig. 2G, H, and I). Surprisingly, we also found that V5-tagged Ago2 or Dcr2 could be detected in myc-tagged Piwi4 pulldown samples (Fig. 2D and E). Again, this was confirmed in reciprocal experiments (Fig. 2J and K). In control experiments, Ago2 and Dcr2 (known to interact in the siRNA pathway) were also found to interact with each other (Fig. 2F and L), as would be expected. In another control experiment, myc-tagged eGFP, unlike Piwi4, showed no association with any of the tested proteins (Fig. 2M to R), indicating that the myc tag and V5 tag did not interact with each other.

### Piwi4 interactions with small RNAs.

It was previously reported that vpiRNAs in Aag2 cells are not produced or bound by Piwi4, despite Piwi4 displaying antiviral activity (13, 27, 28). Similar to studies previously conducted with SINV (27), we found that Piwi5 and Ago3 were required for the production of SFV-specific vpiRNAs (see Fig. S1 in the supplemental material).

10.1128/mSphere.00144-17.1 FIG S1  Effect of Piwi protein knockdown on vpiRNA levels. Aag2 cells transfected with dsRNA against Ago3 (dsAgo3), Piwi5 (dsPiwi5), Piwi6 (dsPiwi6), or eGFP (dseGFP) were infected with SFV4 (at 24 h p.t.) at an MOI of 1, and total RNA was isolated for small RNA analysis at 24 h p.i. The numbers of 24- to 29-nt-long small RNA reads mapping to the SFV genome (red; positive numbers on the *y* axis) or antigenome (green; negative numbers) are shown. Download FIG S1, PDF file, 0.03 MB. Copyright © 2017 Varjak et al. 2017 Varjak et al. This content is distributed under the terms of the Creative Commons Attribution 4.0 International license.

**FIG 3:**
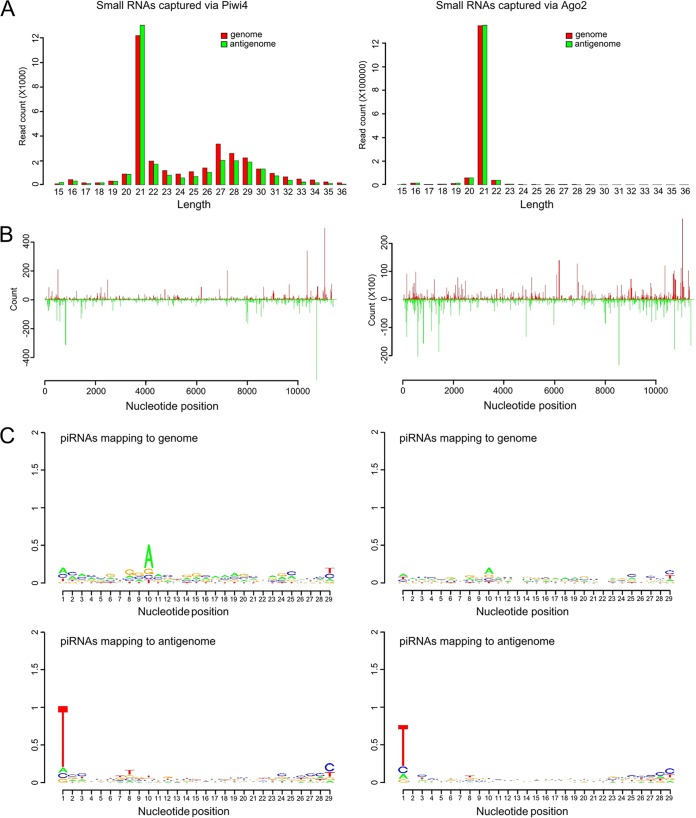
Characterization of SFV-specific small RNAs bound by Piwi4 and Ago2 in Aag2 cells. V5-eGFP-, V5-Piwi4-, or V5-Ago2-expressing cells were infected with SFV at an MOI of 10. At 24 h p.i., V5-tagged proteins were pulled down to further isolate and sequence the protein-bound small RNAs. Properties of Piwi4-bound (left) and Ago2-bound (right) small RNAs are shown. (A) Length distributions (in nucleotides) are shown for small RNAs mapping to the SFV genome (red) or antigenome (green) bound to either Piwi4 or Ago2. (B) Distribution of Ago2- and Piwi4-captured small RNAs of 21 nt in length along the SFV genome (red; positive numbers on the *y* axis) or antigenome (green; negative numbers on the *y* axis). (C) Relative nucleotide frequency and conservation for the position of the 24- to 29-nt SFV-specific small RNAs mapping either to the genome or antigenome. Because a DNA template was used for sequencing, U is represented by T in the sequences. The experiments shown here were repeated independently three times; results of the third experiment are shown.

Due to the associations between Piwi4 and Ago2, as well as Dcr2, which are key effectors of the exo-siRNA pathway, it could be that Piwi4 either directly or indirectly (through Ago2/Dcr2) binds 21-nt-long vsiRNAs instead of or alongside vpiRNAs. To investigate this possibility, V5-tagged Piwi4 was immunoprecipitated from SFV4-infected Aag2 cells (multiplicity of infection [MOI] of 10), and small RNAs associated with Piwi4 were isolated and sequenced. V5-eGFP was used as a negative control in these experiments, and unspecific binding to the beads, antibody, and/or V5-eGFP was found to be negligible compared to material captured by Ago2 or Piwi4 (Table S1). Virus-specific small RNAs of 21 nt in length were enriched in the Piwi4 pulldown samples (Fig. 3A). These findings are comparable with results from pulldown of V5-Ago2 from SFV-infected cells, which also showed enrichment of 21-nt vsiRNAs, as expected (Fig. 3A). However, the number of vsiRNA reads for the Piwi4 pulldown sample was approximately 100 times lower, despite similar expression levels of both tagged proteins (Fig. 3B) and amounts immunoprecipitated (Fig. S3). The distribution profiles of 21-nt-long RNA molecules captured by either Ago2 or Piwi4 were found to be similar along the SFV genome and antigenome (Fig. 3B).

10.1128/mSphere.00144-17.6 TABLE S1  Number of SFV-specific sequencing reads obtained by analyzing small RNA in total cellular RNA samples or in samples that were captured by pulldown of V5-tagged eGFP, Piwi4, or Ago2 from Aag2 or AF319 cells. Download TABLE S1, PDF file, 0.2 MB. Copyright © 2017 Varjak et al. 2017 Varjak et al. This content is distributed under the terms of the Creative Commons Attribution 4.0 International license.

In addition, following reanalysis of the SINV-specific small RNA sequencing data sets recently published by Miesen and colleagues in 2015 (27), we found that Piwi4 is associated predominantly with 21-nt-long SINV-specific vsiRNAs (Fig. S2). This finding had not been originally described and is in agreement with our experimental data. Although it had been stated by Miesen and colleagues that Piwi4 was not associated with vpiRNAs (27), we were able to detect Piwi4-associated SFV-specific small RNAs in the range of 24 to 29 nt (Fig. 3A). They exhibited piRNA-specific features (A_10_ bias for genomic small RNA, U_1_ bias for antigenomic small RNA), although no strand specificity was found. Similar characteristics for vpiRNAs were found for small RNAs captured by Ago2 (Fig. 3C).

10.1128/mSphere.00144-17.2 FIG S2  Piwi4-bound small RNA from SINV-infected cells, based on reanalysis of data previously published by Miesen et al. in 2015 (2). Aag2 cells were first transfected with tagged protein and infected with SINV immediately after transfection at an MOI of 1. The size distribution is shown for small RNAs aligning to SINV-eGFP genome (red) or antigenome (green) captured via V5-tagged Piwi4 (V5-tagged eGFP used as an unspecific control) at 48 h p.i. Download FIG S2, PDF file, 0.03 MB. Copyright © 2017 Varjak et al. 2017 Varjak et al. This content is distributed under the terms of the Creative Commons Attribution 4.0 International license.

10.1128/mSphere.00144-17.3 FIG S3  Immunoprecipitation of V5-tagged protein. Immunoblot analysis of the immunoprecipitation (IP) samples obtained from Aag2 cell lines infected with SFV (MOI, 10). At 24 h p.i., IP of V5-tagged eGFP, Piwi4, or Ago2 was conducted using magnetic beads carrying anti-V5 antibody. Download FIG S3, PDF file, 0.4 MB. Copyright © 2017 Varjak et al. 2017 Varjak et al. This content is distributed under the terms of the Creative Commons Attribution 4.0 International license.

To summarize these results, pulldown of Piwi4 showed the presence of SFV-specific vsiRNAs as well as, to a lesser extent, vpiRNAs. However, due to the interaction of Piwi4 with proteins of the siRNA and the piRNA pathways that are known to bind vsiRNAs (Ago2) and vpiRNAs (Piwi5, Piwi6, and Ago3), it was not possible to conclude with certainty whether the detected small RNAs were bound by Piwi4 or by the interacting proteins, or by both.

### Characterization of Dcr2 KO cells.

Based on our current understanding of the exo-siRNA pathway, Dcr2 is responsible for cleavage of viral dsRNAs into vsiRNAs, which are used by Ago2 for sequence-specific cleavage of the target RNA (1). It is unclear if these vsiRNAs could be loaded into Piwi4 as well as Ago2. In order to determine if the antiviral activity of Piwi4 is dependent on Dcr2-produced vsiRNAs, a gene knockout approach was pursued to create a defined genetic background for further analysis of Piwi4 properties.

The Aag2 cell line was originally generated from a pool of *A. aegypti* embryos (31). To generate the Dcr2 KO cell line, we first sorted single-cell Aag2 suspensions to establish a homogeneous clonal background for CRISPR-Cas9 experiments. The single-cell clone selected for CRISPR-Cas9 experiments (designated AF5) was confirmed to behave similarly to the parental Aag2 cell line in terms of transfection efficiency, infection efficiency with various viruses, and immune status, among other factors (data not shown). We then generated a clonal homozygous Dcr2 KO cell line (designated AF319) by using a guide RNA (gRNA) targeted against exon 1 of the Dcr2 gene. The obtained single-cell colonies were screened for the loss of Dcr2 activity via reporter-based silencing assays, as the Dcr2 KO cells should lack dsRNA-based silencing.

**FIG 4:**
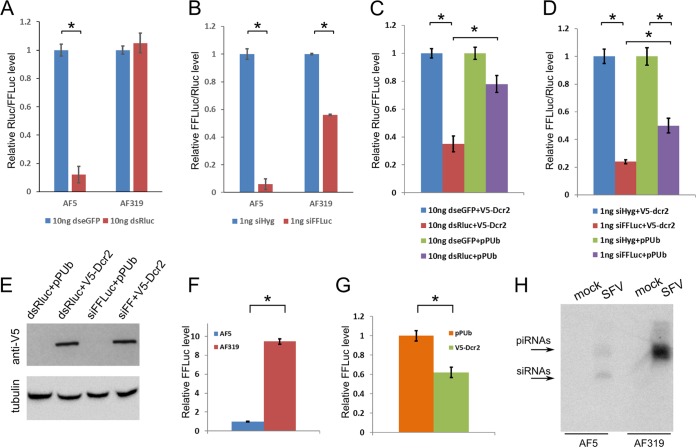
Characterization of a Dcr2 knockout Aag2 cell line. (A) Validation of Dcr2 knockout by cotransfection of FFLuc and Rluc expression constructs together with dsRNA against Rluc (dsRluc) into parental AF5 clone and the derived Dcr2 knockout line, AF319. dsRNA against eGFP (dseGFP) was used as a control. At 24 h p.t., cells were lysed to determine the luciferase levels; relative luciferase levels (Rluc/FFluc; with that with cells transfected with dseGFP set to 1) are shown on the *y* axis. (B) As an alternative silencing inducer, siRNAs against FFLuc (siFFLuc) or hygromycin B resistance gene (siHyg control) were transfected; relative luciferase levels (FFluc/Rluc; with cells transfected with siHyg set to 1) are given on the *y* axis. (C) Repeat of experiments described above, but in AF319 cells, which included in addition to FFLuc and RLuc expression plasmids and dsRNAs (dsRluc or dseGFP) a V5-tagged Dcr2-expressing plasmid (V5-Dcr2) or pPUb (control). (D) Similarly, the effect of expression of V5-Dcr2 in AF319 cells on FFLuc silencing with siRNAs (siFFLuc, siHyg) is shown. (E) Detection of V5-tagged Dcr2 following cotransfection of FFLuc, Rluc, and pPUb or V5-Dcr2 expression plasmids with dsRluc or siFFLuc into AF319 Dcr2 KO cells using anti-V5 antibody. The bottom panel shows Western blot detection of tubulin as the loading control. (F) AF319 Dcr2 KO and parental AF5 cells were infected with SFV(3H)-*FFLuc* at an MOI of 0.01 and lysed at 48 h p.i., and luciferase activity was measured. (G) Effect of expression of Dcr2 in AF319 Dcr2 KO cells on SFV(3H)-*FFLuc* (MOI of 0.01) replication by transfection of pPUb-V5-Dcr2 or pPUb 24 h prior to viral infection; cells were lysed at 48 h p.i. Mean values of three (panels A to E) or four (panels F and G) independent experiments performed in triplicate are presented, with standard errors. *, significant (*P* < 0.05) by Student’s *t* test. (H) Northern blot analysis of small RNA fractions isolated from SFV-infected (MOI of 10, 24 h p.i.) AF5 and AF319 cells. Following size separation, RNAs were probed with a combination of radioactively labeled oligonucleotides to detect siRNAs and piRNAs.

Dcr2 KO cells (AF319) or the parental cells (AF5) were cotransfected with firefly luciferase (FFLuc) and *Renilla* luciferase (Rluc) expression constructs together with either Rluc-specific dsRNA or eGFP-specific dsRNA (control). As expected, using FFLuc levels as an internal control, sequence-specific dsRNA-based reduction of Rluc was detected in the AF5 (parental) cell line compared to the control with dsRNA against eGFP. This was not observed in the AF319 (Dcr2 KO) cells (Fig. 4A). Similar experiments with siRNAs targeting FFLuc showed a sequence-specific reduction in both AF319 and AF5 cells, as expected, as transfection of siRNAs bypasses Dcr2 activity, although reduced silencing efficiency was observed in AF319 cells (Fig. 4B). To further validate the Dcr2 KO cell lines, V5-tagged Dcr2 was transiently reintroduced into these cells (Fig. 4E). The reexpression of Dcr2 restored the sequence-specific dsRNA-based silencing (Fig. 4C and E). In addition, the presence of Dcr2 also increased the effect of siRNA-based silencing (Fig. 4D and E). These results verified the lack of Dcr2 activity in AF319 cells.

Thus far, only the *Aedes albopictus* cell lines C6/36 and C7/10 have been used to describe the effects of nonfunctional Dcr2 on RNAi pathways (12). However, the lack of directly comparable Dcr2-positive cells makes it difficult to draw conclusions. In those two cell lines, vpiRNAs but not vsiRNAs were produced and virus replication usually increased relative to levels in other *A. albopictus* cells. To verify that similar results could be obtained in the Aag2 Dcr2 KO cells produced in this study, AF5 and AF319 cells were infected with SFV expressing FFLuc [SFV(3H)-*FFLuc*] at a low MOI (0.01). SFV had been previously shown to induce the production of vsiRNAs and vpiRNAs in Aag2 cells (7, 13), and knockdown of Ago2 or Piwi4 resulted in increased virus replication. Therefore, it would be expected that the lack of Dcr2 would also enhance virus replication in the AF319 cell line. Indeed, FFLuc expression was significantly increased in AF319 cells compared to AF5 cells at 48 h postinfection (p.i.) (Fig. 4F). Reintroduction of Dcr2 into AF319 cells again reduced virus replication (Fig. 4G) compared to control cells transfected with empty vector. Additionally, the analysis of small RNAs isolated from SFV-infected AF5 and AF319 cells indicated the lack of vsiRNAs in AF319 and much larger amounts of vpiRNAs (Fig. 4H).

**FIG 5:**
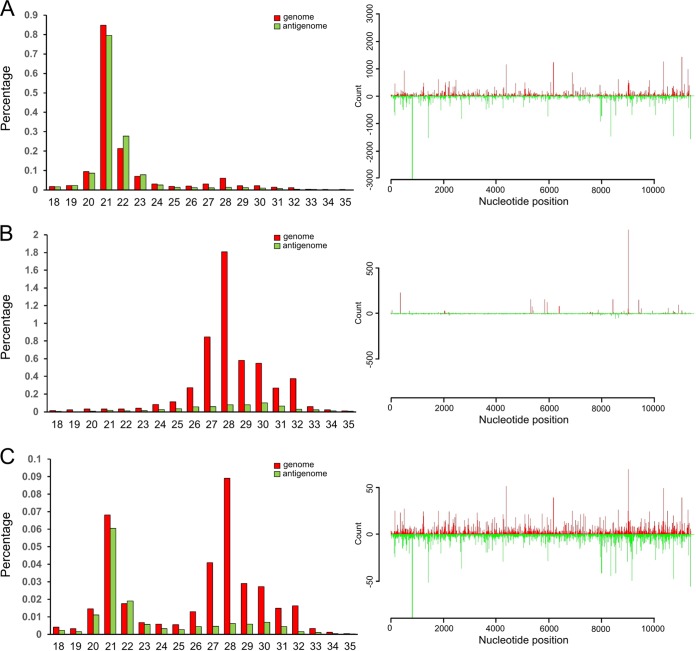
Comparison of SFV-derived small RNAs in Aag2 cells. RNA from SFV4-infected (MOI, 10) parental AF5 (A), Dcr2 KO line AF319 (B), or AF319 cells transfected with the V5-Dcr2 expression construct (C) was isolated at 24 h p.i., and small RNAs were sequenced and characterized. (Left panels) Size distribution of small RNA sequences mapping to the SFV genome (red) or antigenome (green) (as percentages of the total reads). (Right) Distribution of 21-nt small RNAs along the SFV genome (red; positive numbers on the *y* axis) or antigenome (green; negative numbers on the *y* axis).

Sequencing of small RNAs from SFV4-infected Dcr2 KO cells showed that they were incapable of producing 21-nt vsiRNAs, in contrast to the parental AF5 cell line (Fig. 5A and B). Reintroduction of transiently expressed V5-tagged Dcr2 into AF319 cells again allowed production of these vsiRNAs (Fig. 5C), similar to those observed in the AF5 cell line and in previous studies (7, 13). In contrast, vpiRNAs in both AF5 and AF319 cell lines had similar characteristics (Fig. 5A and B; Fig. S4A and B), but the number of vpiRNAs in AF319 cells was much higher, which correlated with increased virus replication in those cells and greater production of vpiRNAs, as visualized by small RNA Northern blot analysis (Fig. 4F and H). Mapping and characterization of small RNAs of 24 to 29 nt to the SFV genome or antigenome in both cell lines resulted in similar distributions as described in previous studies (12, 13, 27), as well as the presence of a ping-pong signature (Fig. S4A and B). Thus, knockout of Dcr2 in Aag2 cells did not alter vpiRNA production, similar to results observed for *A. albopictus* (12). Moreover, reintroduction of Dcr2 in the AF319 cells did not alter vpiRNA production (Fig. 5C; Fig. S4C), as expected. These findings matched data previously reported for C6/36 and C7/10 cells, which are Dcr2 deficient and allow AF5 and AF319 cells to be used for further studies on RNAi in a well-defined Dcr2 KO genetic background with a direct control cell line.

10.1128/mSphere.00144-17.4 FIG S4  Analysis of virus-specific piRNAs from SFV-infected cells. AF5 (parental) (A), AF319 (Dcr2 KO) (B), and AF319 cells transiently expressing V5-Dcr2 cells were infected with SFV at an MOI of 10. At 24 h p.i., we isolated total RNA, which was subjected to small RNA analysis via sequencing. The left panel shows the distribution of 24- to 29-nt small RNAs along the SFV genome (red; positive numbers on the *y* axis) or antigenome (green; negative numbers on the *y* axis). The center panel illustrates relative nucleotide frequencies and conservation along the length of 24- to 29-nt-long small RNAs that mapped to the SFV genome or its antigenome. The logo motifs for piRNAs aligned to the genomic strand are shown in the middle panel and those to the antisense antigenomic strand in the rightmost panel. Download FIG S4, PDF file, 0.6 MB. Copyright © 2017 Varjak et al. 2017 Varjak et al. This content is distributed under the terms of the Creative Commons Attribution 4.0 International license.

### Effect of Piwi4 silencing in Dcr2 KO cells on virus replication.

**FIG 6:**
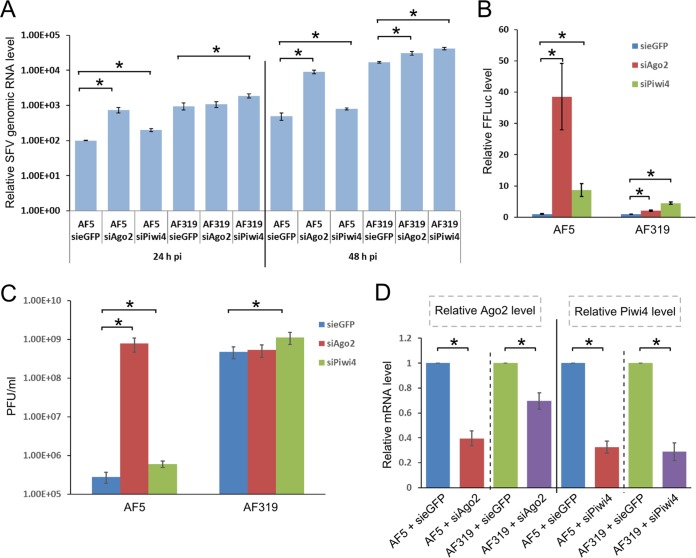
Effects of Piwi4 and Ago2 knockdown on SFV replication in Dcr2 knockout cells. (A) AF319 (Dcr2 KO) cells transfected with gene-specific siRNA (Ago2, Piwi4, or eGFP) were infected with wild-type SFV4 (MOI, 0.01) to measure relative amounts of viral genomic RNA (at 24 h p.i. and 48 h p.i.) by using qRT-PCR. Mean relative RNA levels are shown from four independent experiments in which ribosomal S7 was used as a housekeeping gene; error bars show errors of means. (B) Relative FFluc levels in siRNA-transfected (Ago2, Piwi4, or eGFP as control) cells (Dcr2 KO AF319 cells or parental cell line AF5) at 48 h p.i. with SFV(3H)-*FFLuc* (MOI, 0.01). The mean of three independent experiments performed in triplicate are shown, with standard errors. (C) AF5 or AF319 cells transfected with siRNAs against eGFP, Ago2, or Piwi4 were infected with wild-type SFV (MOI, 0.01), and medium was harvested at 48 h p.i. to measure PFU. Means of 5 independent experiments are shown, and error bars indicate standard deviations. (D) Detection of relative Ago2 and Piwi4 transcript levels in AF5 and AF319 cells by qRT-PCR at 48 h p.t. for siRNAs targeting Ago2, Piwi4, or eGFP (siAgo2, siPiwi4, or sieGFP). *, significant difference (*P* < 0.05) by Student’s *t* test.

The effect of Piwi4 and Ago2 silencing using target-specific siRNAs on SFV replication was assessed by quantitative reverse transcription-PCR (qRT-PCR). As expected, at 24 and 48 h p.i. there was more viral genomic RNA present in AF319 than in AF5 cells (Fig. 6A). Moreover, silencing of Ago2 and Piwi4 resulted in 10- and 2-fold increases, respectively, in viral RNA levels in AF5 cells. In AF319 cells, at 24 h and 48 h p.i. we found that knockdown of Piwi4 benefitted the virus, and approximately 2-fold increases in the amounts of genomic RNA were detected. However, silencing of Ago2 resulted in a slight increase of viral RNA loads by 48 h p.i. (Fig. 6A). We verified the effect of Piwi4 or Ago2 silencing in Aag2 Dcr2 KO AF319 cells on virus by using SFV(3H)-*FFLuc*. It was found that Ago2 knockdown in Dcr2 KO cells had a significantly reduced effect on virus replication (as determined by FFLuc expression) compared to the AF5 parental cells (2-fold increase versus 40-fold increase) (Fig. 6B). These data support the hypothesis that efficient antiviral activity of Ago2 is dependent on Dcr2-produced vsiRNAs. In contrast, Piwi4 silencing resulted in similar increases of luciferase expression in both Dcr2 KO cells and the parental cell line (5-fold and 9-fold, respectively) compared to the negative control. Similarly, if siRNA-transfected cells were infected with SFV4 at an MOI of 0.01 and virus production was measured at 48 h p.i., it was found that Ago2 silencing boosted virus production 1,000-fold in AF5 cells but had no effect in AF319 cells (Fig. 6C). In contrast, knockdown of Piwi4 resulted in three times more virus production, regardless of which cell line was used (*t* test, *P* < 0.05). However, it should be noted that siRNA-based knockdown of Ago2 was less effective in Dcr2 KO cells than in the parental cell line (Fig. 6D). In summary, these results suggest that unlike Ago2, Piwi4 has antiviral activity against SFV and functions independently of Dcr2.

**FIG 7:**
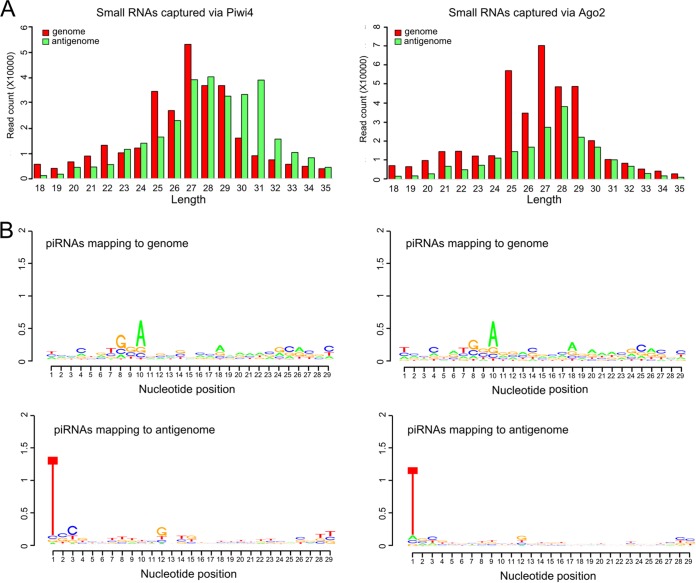
SFV-specific small RNAs bound by Piwi4 or Ago2 in SFV-infected AF319 cells**.** V5-eGFP-, V5-Piwi4-, or V5-Ago2-expressing AF319 cells were infected with SFV at an MOI of 10. At 24 h p.i., V5-tagged protein was pulled down and RNA was isolated in order to characterize protein-associated small RNAs. Characteristics of V5-Piwi4-captured (left) and V5-Ago2-captured (right) small RNAs are shown. (A) Length distributions of small RNAs that align to the SFV genome (red) or antigenome (green). (B) Relative nucleotide frequencies and conservation for the positions of the 24- to 29-nt-long SFV-specific small RNAs that align to the genome or antigenome. As DNA was used as the template for sequencing, T represents U. Experiments shown were repeated twice independently. Results of the second series of experiments are shown.

As Piwi4 retained its antiviral activity in a Dcr2 KO background, we could not exclude the possibility that Piwi4-associated 21-nt small RNAs (Fig. 3) are produced independently of Dcr2, for example, via the microRNA processing pathway. To test this, AF319 cells stably expressing V5-tagged Piwi4, Ago2, or eGFP (control) (Fig. S5A) were created as described above (Fig. 1). AF319 cell lines were infected with SFV4 (MOI, 10), and at 24 h p.i. V5-tagged proteins were immunoprecipitated (Fig. S5B) and RNA was isolated and sequenced. Analysis of captured RNA showed that both Piwi4 and Ago2 pulled out predominantly small RNAs that were longer than 21 nucleotides (Fig. 7A; Table S1). In addition, these longer RNAs had the characteristics of piRNAs (A_10_ bias for genomic RNAs and U_1_ bias for antigenomic RNAs) (Fig. 7B), but no strand bias could be detected. These data show that the absence of Dcr2 results in the disappearance of vsiRNAs associated with Ago2 and Piwi4, and more importantly, that Piwi4-associated 21-nt-long RNAs in normal cells are not of piRNA or miRNA origin.

10.1128/mSphere.00144-17.5 FIG S5  Stable expression of tagged proteins in AF319 cells. (A) Immunoblot analysis of AF319 cells that express V5-tagged eGFP, Piwi4, or Ago2. The cell lines were constructed as described for Fig. 1. Tubulin was used as a loading control. (B) Cells expressing V5-tagged eGFP, Piwi4, or Ago2 were infected with wild-type SFV, and 24 h p.i. these proteins were immunoprecipitated (IP) using anti-V5 tag antibodies bound to magnetic beads. Next, samples were probed for the V5 tag. Download FIG S5, PDF file, 0.9 MB. Copyright © 2017 Varjak et al. 2017 Varjak et al. This content is distributed under the terms of the Creative Commons Attribution 4.0 International license.

**FIG 8:**
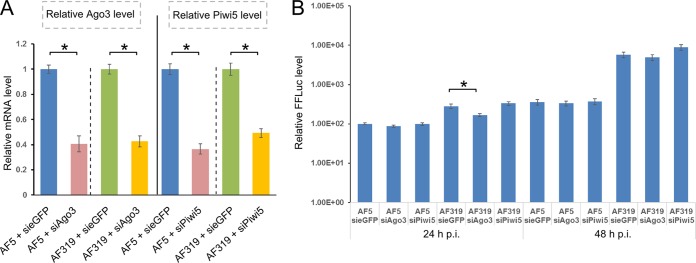
Effects of silencing Ago3 and Piwi5 on SFV replication. (A) Relative Ago3 and Piwi5 transcript levels in AF5 and AF319 cells, as determined by qRT-PCR 24 h p.t., of siRNAs targeting Ago3, Piwi5, or eGFP (siAgo3, siPiwi5, or sieGFP). (B) Transfected cells were infected with SFV(3H)-*FFLuc* (MOI, 0.01) to determine luciferase expression levels at 24 h and 48 h p.i. Means (on a log scale) are from four independent experiments conducted in triplicate, and standard errors are shown. *, significant difference (*P* < 0.05) by Student’s *t* test.

To verify that the fraction of piRNAs still associated with Piwi4 could play a role in its antiviral activity, Ago3 or Piwi5 expression was silenced in AF5 and AF319 cells by transfection of corresponding siRNAs. Following this, cells were infected with SFV(3H)-*FFluc* at an MOI of 0.01. In AF5 cells, silencing of Ago3 or Piwi5 had no effect on virus replication at 24 h and 48 h p.i. (Fig. 8), as shown previously in Aag2 cells (13). However, despite higher production levels of virus-specific piRNAs in AF319 cells, silencing of Ago3 and Piwi5 had no enhancing effect on SFV replication (Fig. 8), further indicating that the antiviral effects of Piwi4 are independent of piRNA production.

## DISCUSSION

Production of virus-specific vsiRNAs and vpiRNAs in mosquitoes or derived cells has been demonstrated for all major RNA arbovirus families (1). However, due to technical and practical issues, many such studies on insect antiviral RNAi responses were initially conducted in arbovirus-infected *D. melanogaster* flies or their derived cells (32–37).

Due to differences in the piRNA pathway between flies and mosquitoes (12, 13, 25), the fruit fly model does have limitations, and this becomes more evident when studying the piRNA pathway. Although little is known about the piRNA pathway in mosquitoes, the involvement of the different PIWI proteins, especially in the production of vpiRNAs and the potential antiviral activities of vpiRNAs, is an area of intense research. Recently, Piwi5 (and to a lesser extent Piwi6) and Ago3 have been identified as being required for the production of SINV- and DENV-specific vpiRNAs in Aag2 cells (27, 28), thereby indirectly supporting Dcr2-independent production of vpiRNAs (in contrast to vsiRNAs). This is further strengthened by the fact that naturally Dcr2-deficient cells (*A. albopictus*-derived C6/36 and C7/10 cell lines) or Dcr2 KO cells, as described here, are unable to produce vsiRNAs but do produce vpiRNAs (9, 11, 12, 14) (Fig. 5; Fig. S4).

There is no direct evidence that vpiRNAs display antiviral activity and/or supplement the role of vsiRNAs. In the case of DENV, silencing of Ago3 decreased viral genomic RNA amounts in infected Aag2 cells (28), and for orthobunyaviruses, silencing of Ago3, Piwi5, or Piwi6 did not enhance virus replication; more specifically, silencing of Piwi5 had a negative effect on Schmallenberg virus (38). For SFV infection of Aag2 cells, no increase in viral replication has been reported when Piwi5 or Ago3 is silenced, although these proteins are important in the production of SFV-specific vpiRNAs (Fig. S1) (13). Furthermore, silencing of Ago3 and Piwi5 did not enhance virus replication in AF319 cells, despite the larger amounts of piRNAs present in the cells (Fig. 4, 5, and 8). In contrast, the knockdown of Piwi4 resulted in increased virus replication but had no effect on vpiRNA production (13, 27, 28). It has been speculated that Piwi4 binds vpiRNAs and may have a direct effector function. However, further analysis of Piwi4 in SINV-infected cells did not support this hypothesis, thus posing the question of how Piwi4 acts in an antiviral manner (27, 28).

To understand the roles and properties of Piwi4 better, we studied the interactome of Piwi4 by using cell lines that stably express a tagged protein of interest. To obtain these cell lines, we developed a novel and very potent approach that can be used in mosquito cells, as classical approaches used in mammalian cells are not suitable (unpublished observations). Coimmunoprecipitation experiments showed that Piwi4 is associated with members of both the exo-siRNA (Dcr2, Ago2) and piRNA (Piwi5, Piwi6, Ago3) pathways (Fig. 2). This suggests that Piwi4 either bridges these pathways, shuttles between them, or acts in a separate pathway in the cells. Further studies are required to understand whether interactions between Piwi4 and piRNA or siRNA pathway proteins are direct or indirect, e.g., occurring via a protein or RNA bridge.

The associations of Piwi4 with both pathways were further supported by the detection of SFV-specific vsiRNAs and vpiRNAs, although the majority were 21-nt vsiRNAs. In addition, the smaller number of Piwi4-associated vpiRNAs showed no strand specificity (Fig. 3). Reanalysis of recently published small RNAs bound to Piwi4 in SINV-infected cells underlined the association of Piwi4 with 21-nt vsiRNAs (Fig. S2). It has to be noted that vpiRNAs were not found to be associated with Piwi4 in a previous study (27). At present, it is not known if the vsiRNAs and piRNAs we identified were bound by Piwi4 or a Piwi4-interacting protein, such as Ago2, Piwi5, or Ago3; the data support the latter option. vsiRNAs identified in Ago2 pulldown samples resulted in 100 times more reads than were found in Piwi4 pulldown samples, despite comparable amounts of tagged proteins being expressed in cells and similar vsiRNA profiles (Fig. 1 and 3B; Fig. S3). This difference may be due to the fact that Ago2 has a higher affinity for vsiRNAs than Piwi4, or that supposedly Piwi4-bound vsiRNAs are in fact bound by Ago2, which forms complexes with Piwi4. If the antiviral activity of Piwi4 is related either to its association with proteins of the siRNA pathway or binding vsiRNAs, it would be expected that Piwi4 antiviral activity is lost in Dcr2 knockout cells. Knockdown of Piwi4 in Dcr2 KO cells still resulted in an increase of SFV replication (Fig. 6), although Piwi4 lost its association with vsiRNAs (Fig. 7). In contrast, similar experiments with Ago2 showed an almost complete loss of its antiviral properties in Dcr2 KO cells (Fig. 6), and no siRNAs were found to bind to Ago2. This supports the findings that Ago2 is dependent on Dcr2 for its antiviral activity, while Piwi4 is not.

No strand specificity could be found for Piwi4-associated vpiRNAs. Moreover, silencing of Ago3 and Piwi5 (Fig. S1) did not have an enhancing effect on SFV in Aag2 cells (13) or Dcr2-deficient cells (Fig. 8). In addition, SFV replicated better in AF319 cells. Altogether, these data show that piRNAs do not have antiviral activities, and this refutes the idea that piRNAs can replace siRNAs in the control of virus infections in cells lacking Dcr2. Thus, we speculate that Piwi4 is a noncanonical effector and may mediate an antiviral function(s) independently of either pathway.

The exact mechanism of action of Piwi4 remains to be elucidated, and it could be related to translational control or specific motif recognition. The expanded set of PIWI proteins in mosquitoes (13, 25) opens up the possibility of redundancy and also divergence, which seems to be the case with Piwi4. Further studies are needed to identify and map all interactions between piRNA and siRNA pathway proteins and to verify whether there are any dynamic changes during infection. In addition, the publication of the *A. albopictus* genome (39) invites the possibility for comparative studies between two important vector mosquito species. Overall, these results demonstrate the multifaceted interaction between the siRNA and piRNA pathways in *A. aegypti* and identify Piwi4 as a noncanonical PIWI protein that forms a complex with members of the siRNA and piRNA pathways, but with Piwi4-mediated antiviral activities potentially independent of either pathway.

## MATERIALS AND METHODS

### Cells.

*A. aegypti*-derived Aag2 cells (obtained from P. Eggleston, Keele University, United Kingdom) were grown in L-15 medium plus Glutamax (Life Technologies, Inc.) supplemented with 10% tryptose phosphate broth (TPB; Life Technologies, Inc.), 10% fetal bovine serum (FBS; Life Technologies, Inc.), and penicillin-streptomycin (final concentrations of 100 units/ml and 100 µg/ml, respectively; Life Technologies, Inc.). For cell lines expressing tagged proteins, zeocin was added to a final concentration of 100 µg/ml (Life Technologies, Inc.), and cells were grown at 28°C. *A. aegypti*-derived Dcr2 KO cells (AF319) and the parental cell line, AF5, were grown at 28°C in L-15 medium supplemented with 10% nonessential amino acids (Life Technologies, Inc.), 10% TPB, 20% FBS, and penicillin-streptomycin (final concentrations of 100 units/ml and 100 µg/ml, respectively). Baby hamster kidney (BHK-21) cells (40) were grown in Glasgow minimum essential medium (Life Technologies, Inc.) supplemented with 10% TPB, 10% newborn calf serum (Life Technologies, Inc.), and penicillin-streptomycin (final concentrations of 100 units/ml and 100 µg/ml, respectively) at 37°C, 5% CO_2_.

### Plasmids.

The firefly luciferase (FFLuc) and *Renilla* luciferase (Rluc) expression plasmids pIZ-Fluc and pAcIE1-Rluc have been previously described (41). The backbone used to generate the remainder of the expression constructs was a plasmid expressing FFLuc under control of the polyubiquitin promoter (PUb) (30), available from Addgene (catalog number 52891). The FFLuc coding sequence was removed, and the resulting plasmid was thereafter named pPUb. Gene sequences were obtained by extracting total RNA from Aag2 cells and synthesizing cDNA. Gene-specific primers were used to amplify the target gene of interest. The zeocin resistance gene was amplified by PCR from pIB/V5-His (Life Technologies, Inc.). A double-2A element from *Thosea asigna* virus was synthesized at Geneart, and tag sequences (myc or V5 tag) were added by PCR. All PCRs were carried out with KOD polymerase (Merck Millipore), and sequences of finalized constructs were verified via Sanger sequencing. The sequences are available upon request.

### Production of stable cell lines.

In order to produce stable cell lines, approximately 3 × 10^6^ cells were transfected with 10 µg of the appropriate plasmid linearized with NotI using Lipofectamine LTX Plus reagent (Life Technologies, Inc.), following the manufacturer’s protocol. At 6 h posttransfection (p.t.), the growth medium was replaced and 48 h later it was again replaced with fresh medium, this time containing zeocin as a selective marker. The medium was continually replaced every 3 or 4 days to remove dead cells; all cell lines were polyclonal.

### Production of the Aag2 Dcr2 knockout cell line AF319.

A gRNA (TAGCAAAATTTAATCGTGCTAGG) targeting exon 1 of the Dcr2 gene was cloned into the *Drosophila* CRISPR vector pAc-sgRNA-Cas9 (42), which was a gift from Ji-Long Liu (Addgene plasmid 49330). This plasmid expresses gRNAs under control of the *Drosophila* U6 promoter and a human codon-optimized N-terminally FLAG-tagged *Streptococcus pyogenes* Cas9 enzyme linked to the puromycin resistance gene via a T2A polyprotein self-cleavage site under control of the *Drosophila* actin 5C promoter. The single-cell clone of Aag2 AF5 cells was transfected with the gRNA-containing plasmid by using Lipofectamine 2000 (Life Technology) at 2.5 µg plasmid per 2 × 10^6^ cells. After 72 h, cells expressing the Cas9 construct were selected by using puromycin (24 µg/ml) for 5 days. Cells were then sorted into single-cell suspensions on a BD FACSAria II flow cytometry sorter (BD Biosciences, Franklin Lakes, NJ, USA) into 200 µl medium in 96-well plates using a 100-µm nozzle and a sheath pressure of 35 lb/in^2^. Clones were expanded and screened for RNAi deficiency. The final clone selected and used throughout the studies was designated AF319.

### Viruses.

SFV4 and SFV4(3H)-*FFLuc* virus stocks were prepared in BHK-21 cells. All virus titrations were done by plaque assay as described previously (13, 43). The *FFLuc* gene was inserted into a duplicated nsP2 cleavage sites at the C terminus of nsP3 and was separated from the nonstructural proteins by the protease activity of nsP2, as described previously (43, 44).

### Protein immunoprecipitation.

For small RNA capture assays, 10^7^ cells stably expressing V5-eGFP, V5-Ago2, or V5-Piwi4 were infected with SFV4 at an MOI of 10. At 24 h p.i., cells were scraped and collected into a 50-ml tube and spun down, followed by washing the cells with phosphate-buffered saline (PBS). Thereafter, the cells were resuspended in 1 ml of lysis buffer (150 mM NaCl, 5 mM MgCl_2_, 20 mM HEPES [pH 7.4], 0.5% Triton X-100, protease inhibitor cocktail [Roche]) prior to transfer into 1.5-ml tubes. Cells were kept on ice for 20 min, followed by centrifugation at 15,000 × *g* at 4°C for 25 min. The supernatant was then transferred into fresh tubes on ice, and mouse anti-V5 (1:500; AB27671; Abcam, Inc.) was added to the supernatant. Tubes were rotated for 2 h at 4°C. Following this, 30 µl of protein G-coated magnetic beads (Dynabead protein G; Life Technologies, Inc.) was added per sample after the beads had been equilibrated with cold washing buffer (150 mM NaCl, 5 mM MgCl_2_, 20 mM HEPES [pH 7.4], 0.5% Triton X-100) immediately prior to addition. Tubes were then again rotated for 1 h at 4°C. By using a magnetic rack, beads were collected, the supernatant was removed, and 500 µl washing buffer was added. Tubes were then returned to rotate for 5 min. This was repeated a further 3 times. The washed beads were finally resuspended in 100 µl of washing buffer, and 1/20 of the volume was subjected to Western blot analysis while the remainder was used for RNA extraction.

For coimmunoprecipitation experiments, 10^7^ cells stably expressing the protein of interest were transfected with 30 µg of plasmid expressing myc-tagged protein under control of the PUb promoter and by using Lipofectamine LTX transfection reagent. The transfection medium was then replaced with fresh medium after 6 h. At 48 h p.t., immunoprecipitation was carried out as described above with the exception that mouse anti-myc (1:500; catalog number 2276; Cell Signaling) was used. After the final wash, beads were resuspended in 100 µl of sample buffer (25 µl of 4× Bolt lithium dodecyl sulfate sample buffer, 10 µl of 10× Bolt reducing agent [Life Technologies, Inc.], 65 µl of H_2_O) and boiled at 95°C for 10 min before samples were analyzed by Western blotting.

### Extraction of protein-bound small RNA.

For extraction of protein-bound small RNA, 5 μl of proteinase K (20 mg/ml) was added to the magnetic beads after they were resuspended in washing buffer, and the samples were placed into a water bath at 37°C for 30 min. Following this, 1 ml of Trizol reagent (Life Technologies, Inc.) was added to the sample and processed according to the manufacturer’s instructions.

### Total RNA extraction and cDNA synthesis.

Trizol (500 µl) was added to 1.8 × 10^5^ AF5 or AF319 cells per well of a 24-well plate. Material from two wells was then pooled, and total cellular RNA was extracted as per the manufacturer’s instructions. Total RNA (1.5 µg) was used for cDNA synthesis using SuperScript III and oligo(dT)_15_ primer (Promega) as previously described (13).

### Small RNA sequencing and sequence analysis.

Cells (1 × 10^6^) were lysed in 1 ml Trizol, and total RNA was extracted according to the manufacturer’s protocol. To increase small RNA precipitation efficiency, glycogen was added as a carrier. Small RNAs of 15 to 40 nt in length were gel purified and sequenced on an Illumina HiSeq apparatus. Data were analyzed as previously described (13, 45).

### qRT-PCR.

Quantitative RT-PCR for Piwi4, Ago2, and the housekeeping gene S7 was performed using specific primers (Table S2), SYBR green master mix (ABI), and an ABI7000 Fast system according to the manufacturer’s protocol. Results were analyzed using the ΔΔ*C*_*T*_ method.

10.1128/mSphere.00144-17.7 TABLE S2  Sequences of DNA oligonucleotides and siRNAs used in the study. Download TABLE S2, PDF file, 0.3 MB. Copyright © 2017 Varjak et al. 2017 Varjak et al. This content is distributed under the terms of the Creative Commons Attribution 4.0 International license.

### Small RNA Northern blot analysis.

Northern blot analysis was conducted as described elsewhere (46). In short, the small RNA fraction was isolated from mock- or SFV-infected cells (MOI of 10, at 24 h p.i.) by using the mirVana miRNA isolation kit (Thermo Fisher Scientific). RNA was size fractioned on a 0.5× TBE–7 M urea–15% polyacrylamide gel, transferred to Hybond NX nylon membranes (GE Healthcare), and chemically cross-linked by using 1-ethyl-3-(3-dimethylaminopropyl) carbodiimide (Sigma). Small RNAs were probed with a set of DNA oligonucleotides (Table S2) that were 5′ end-labeled with [γ-^32^P]ATP (Perking-Elmer) using T4 polynucleotide kinase (New England Biolabs). Hybridization to the oligoprobes was performed overnight at 42°C in Ultrahyb Oligo hybridization buffer (Thermo Fisher Scientific). Membranes were washed twice at 42°C with each of the following three buffers: 2× SSC (1× SSC is 0.15 M NaCl plus 0.015 M sodium citrate) and 0.5% SDS, 2× SSC and 0.2% SDS, and 0.2× SSC and 0.1% SDS. The membrane was exposed to a phosphorimager screen for signal detection.

### Immunoblot analysis.

Protein extracts in sample buffer were separated on Bolt 4%-to-12% bis-Tris Plus gels (Life Technologies, Inc.) and transferred to Protran 0.45-μm nitrocellulose membranes (GE Healthcare Life Sciences) by using the Trans-blot SD semidry transfer cell (Bio-Rad). Membranes were blocked for a minimum of 1 h in 5% (wt/vol) nonfat dry milk in Tween-PBS (PBS with 0.1% Tween 20). Following this, the membrane was incubated for 16 h at 4°C in 2% (wt/vol) nonfat dry milk in Tween-PBS containing either mouse anti-myc (1:1,000 dilution; catalog number 2276; Cell Signaling), mouse anti-V5 (1:2,000, AB27671; Abcam, Inc.), or mouse anti-tubulin (1:5,000; T5168; Sigma-Aldrich). Thereafter, membranes were washed three times for 10 min with Tween-PBS and incubated for an hour in 2% (wt/vol) nonfat dry milk in Tween-PBS with anti-mouse secondary antibody conjugated with horseradish peroxidase (A16072; Life Technologies, Inc.). This was followed by three 10-min washes with Tween-PBS buffer, and enhanced chemiluminescence was used for signal detection (Thermo Fisher Scientific).

### Transfection of nucleic acids.

For coimmunoprecipitation experiments, DNA was transfected using Lipofectamine LTX with Plus reagent (Life Technologies, Inc.) according to the manufacturer’s protocol, with 1 µl of LTX reagent and 0.5 Plus reagent per 1 µg DNA.

For small RNA sequencing analysis of knockdown cells, 1-µg amounts of dsRNAs against Ago3, Piwi5, Piwi6, or eGFP were transfected into 1 × 10^6^ Aag2 cells using Lipofectamine 2000 as described previously (13).

For other experiments, 2 µl of DharmaFECT 2 (GE Healthcare) was used per well of a 24-well plate. For RNAi reporter assays, 10 ng of dsRNA (Rluc specific or eGFP specific) or 1 ng siRNA (targeting FFLuc or hygromycin B resistance gene) was cotransfected with 60 ng of pIZ-Fluc and 100 ng pAcIE1-Rluc; when required, 500 ng of pPUb-V5-Dcr2 or empty pPUb was also included. For silencing of Ago2, Ago3, Piwi4, or Piwi5, 20 pmol of siRNA was used per well, with siRNA targeting eGFP used as the control. For siRNA sequences, see Table S2.

### dsRNA production.

T7 promoter-flanked PCR products of Rluc or eGFP (for primers, see Table S2) were used for *in vitro* transcription. These products were treated with Dnase I and RNase A, followed by column purification of the dsRNA using the RNAi Megascript kit (Thermo Fisher Scientific) as per the manufacturer’s instructions.

### Accession number(s).

The data are available in the NCBI Sequence Read Archive under accession no. PRJNA383671.
