# Supplementary figures and images for: Aedes aegypti Piwi4 Is a Noncanonical PIWI Protein Involved in Antiviral Responses
Source: mSphere. 2017 May 3;2(3):e00144-17. doi: 10.1128/mSphere.00144-17 (PMC5415634; doi:10.1128/mSphere.00144-17)

Supplementary Figure 1

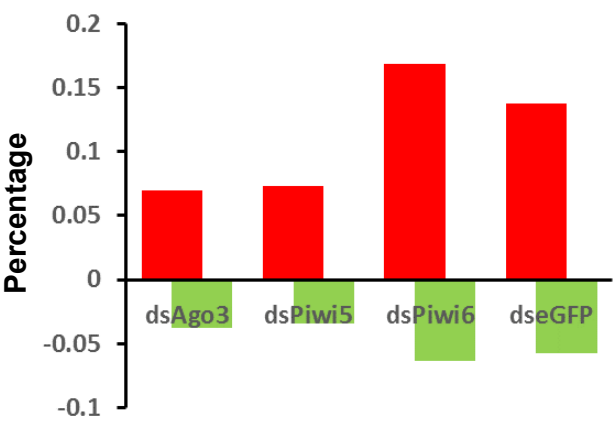

Supplement: FIG S1 [file sph003172280sf1.pdf]

Supplementary Figure 2

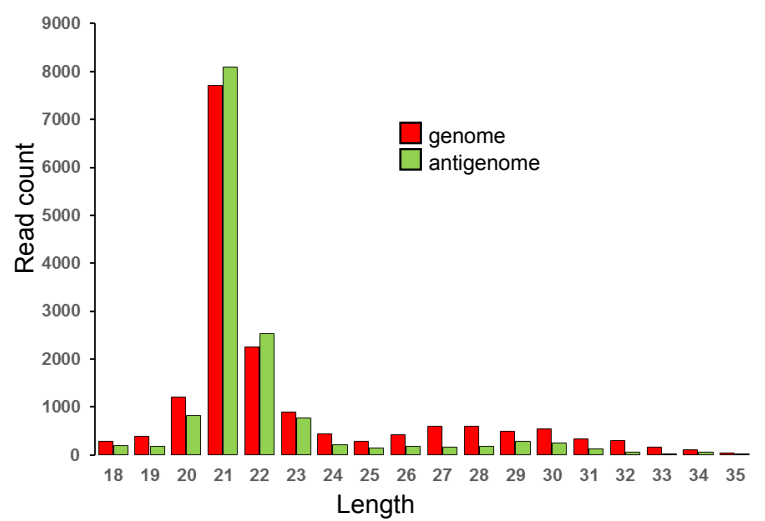

Supplement: FIG S2 [file sph003172280sf2.pdf]

Supplementary Figure 3

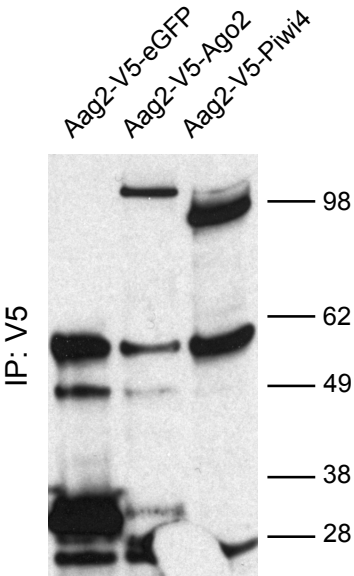

Supplement: FIG S3 [file sph003172280sf3.pdf]

Supplementary Figure 4

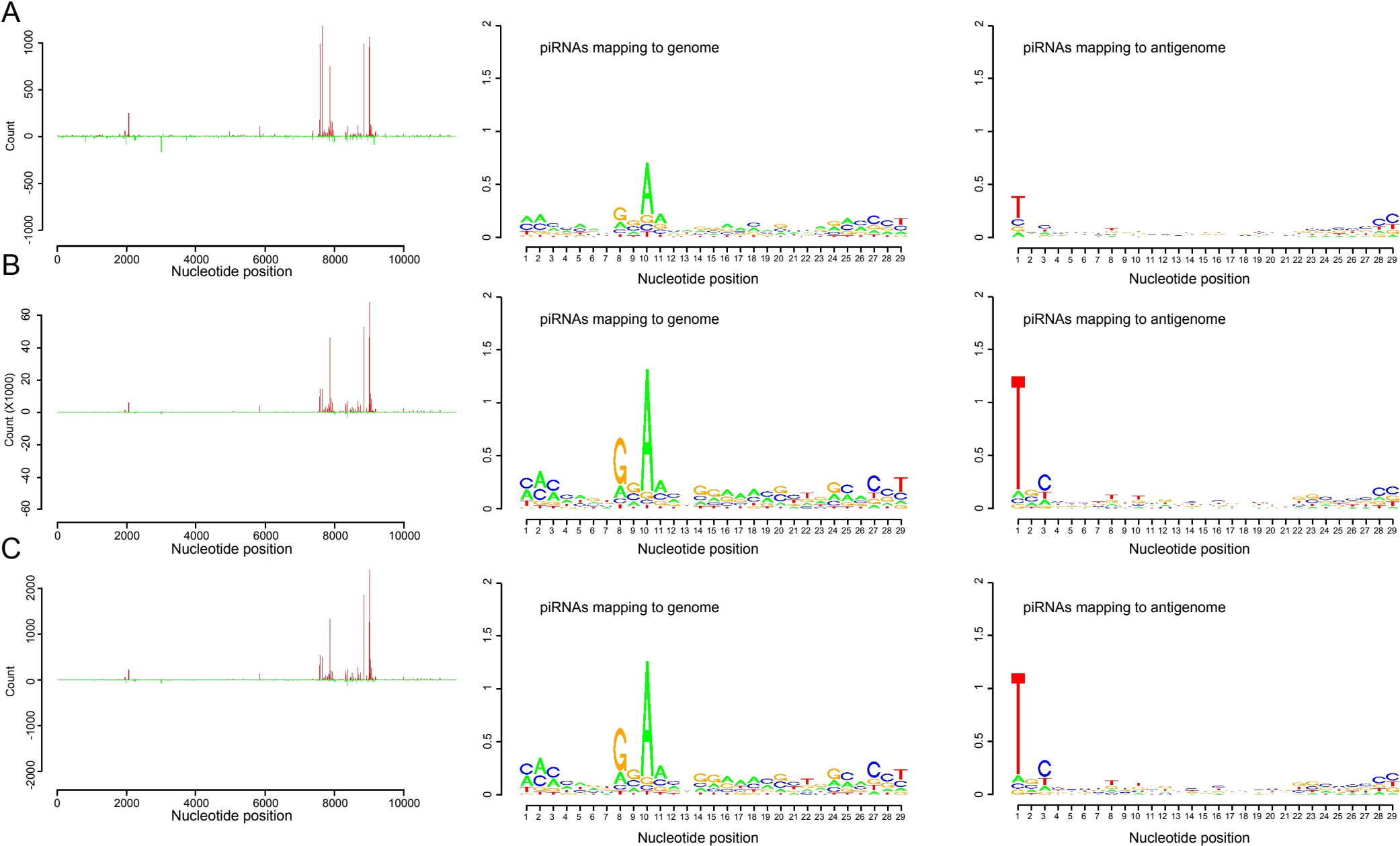

Supplement: FIG S4 [file sph003172280sf4.pdf]

Supplementary Figure 5

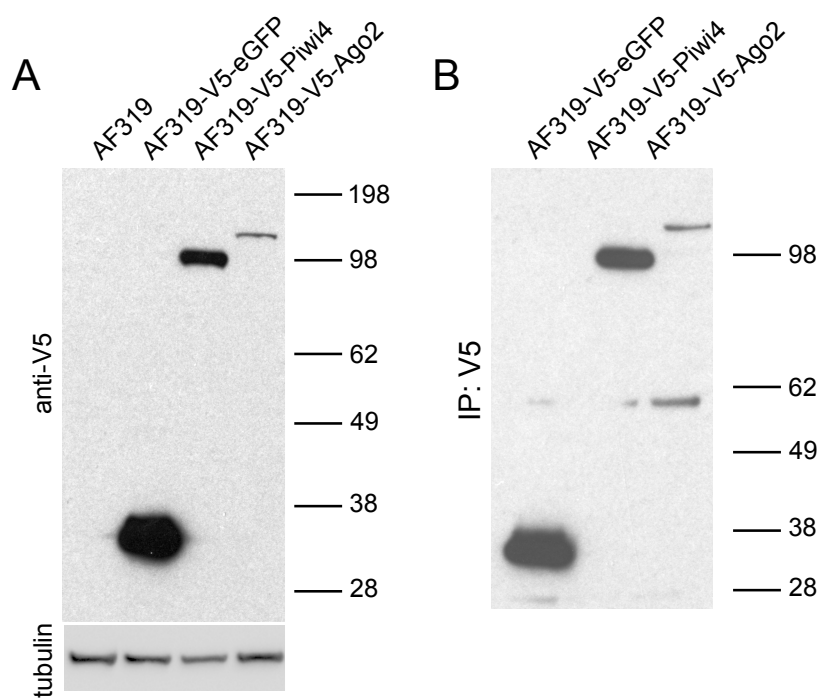

Supplement: FIG S5 [file sph003172280sf5.pdf]
