# Supplementary material for: Aedes aegypti Piwi4 Is a Noncanonical PIWI Protein Involved in Antiviral Responses
Source: mSphere. 2017 May 3;2(3):e00144-17. doi: 10.1128/mSphere.00144-17 (PMC5415634; doi:10.1128/mSphere.00144-17)
Supplement: TABLE S1 [file sph003172280st6.pdf]

Small RNA captured from Aag2 cells			
**First experiment**			
**Pulled down protein**	**Number of SFV-specific 21 nt long reads**	**Number of SFV-specific 24-29 nt long reads**	**Number of total reads**
V5-eGFP	864	260	9523389
V5-Ago2	2703344	27677	11953099
V5-Piwi4	25287	19688	10643535
**Second experiment**			
**Pulled down protein**	**Number of SFV-specific 21 nt long reads**	**Number of SFV-specific 24-29 nt long reads**	**Number of total reads**
V5-eGFP	23477	5489	9095349
V5-Ago2	2390602	26724	13080203
V5-Piwi4	70721	19628	12876943
**Third experiment**			
**Pulled down protein**	**Number of SFV-specific 21 nt long reads**	**Number of SFV-specific 24-29 nt long reads**	**Number of total reads**
V5-eGFP	1310	796	9886415
V5-Ago2	1802931	76621	14347528
V5-Piwi4	41611	25980	7804137
**Small RNA captured from AF319 cells**			
**First experiment**			
**Pulled down protein**	**Number of SFV-specific 21 nt long reads**	**Number of SFV-specific 24-29 nt long reads**	**Number of total reads (>18 nt)**
V5-eGFP	22710	192791	23950572
V5-Ago2	22872	330607	30996905
V5-Piwi4	11307	342123	28544034
**Second experiment**			
**Pulled down protein**	**Number of SFV-specific 21 nt long reads**	**Number of SFV-specific 24-29 nt long reads**	**Number of total reads (>18 nt)**
V5-eGFP	4198	11517	8039205
V5-Ago2	21132	401216	26202139
V5-Piwi4	13502	365319	27606621
**Analysis of total cellular RNA from SFV-infected AF5 and AF319 cells**			
**Cell line**	**Number of SFV-specific 21 nt long reads**	**Number of SFV-specific 24-29 nt long reads**	**Number of total reads**
AF5	189163	31702	11498872
AF319	6780	571825	14139067
AF319+ V5-Dcr2	12823	21043	9956581
**Analysis of total cellular RNA from SFV-infected Aag2 cells if eGFP, Ago3, Piwi5 or Piwi6 had been knocked down**			

Target	Number of SFV-specific 21 nt long reads	Number of SFV-specific 24-29 nt long reads	Number of total reads
eGFP	165519	28655	14678239
Ago3	147053	14371	13351695
Piwi5	191039	16710	15534765
Piwi6	107854	23322	10064479
