# Supplementary material for: Aedes aegypti Piwi4 Is a Noncanonical PIWI Protein Involved in Antiviral Responses
Source: mSphere. 2017 May 3;2(3):e00144-17. doi: 10.1128/mSphere.00144-17 (PMC5415634; doi:10.1128/mSphere.00144-17)
Supplement: TABLE S2 [file sph003172280st7.pdf]

**Primers for making dsRNA, T7 promoter underlined and in italic.**	
**Target**	**Forward/ Reverse primer**
Ago3	*GTAATACGACTCACTATAGGG* GAGTTACCTCATCAATGACGCG/ *GTAATACGACTCACTATAGGG* GGCACTTAAATCCTGTAGGTACCTT
Piwi5	*GTAATACGACTCACTATAGGG* CGTAATGTTGCTGTTTCGAATG/ *GTAATACGACTCACTATAGGG* GGATGTTACCCCTAGGCTAGG
Piwi6	*GTAATACGACTCACTATAGGG* ACCAGAAATAGTGCAAACCCG/ *GTAATACGACTCACTATAGGG* CATGTCGGTTGATAAGGTTGAA
eGFP	*GTAATACGACTCACTATAGGG* GGCGTGCAGTGCTTCAGCCGC/ *GTAATACGACTCACTATAGGG* GTGGTTGTCGGGCAGCAGCAC
**Primers for qRT-PCR**	
**Target**	**Forward/ Reverse primer**
SFV	ATCCAACCTACGCCACAAAC/ GCCGAGTCTTCCCTCAGTC
Ago2	GGCTGCTCACCCAATGTATCAAGA/ AACCGTTCGTTTTGGCGTTGAT
Ago3	GCTTCGTTGAATGGTGACTACAC/ TGACAGTTTGCCTTCTGGTAAG
Piwi5	GAAGTTGAAATAACCACCAAAGAG/ CCATCTACTACGCCGACTTTTC
Piwi4	CTTCTCCACCACAGCCAATG/ GTCCAATCTGCCTGTTCTCCA
S7	CCAGGCTATCCTGGAGTTG/ GACGTGCTTGCCGGAGAAC
**Sense sequence of siRNA**	
**Target**	**Sequence**
Ago2	GAACAAACAACUUCGGUAUUU
Piwi4	GUCAGAAGCACGAUCGUAAUU
Ago3	CUGCAAGGGUGUCGCAAAUUU
Piwi5	CCGCGGAGGUGUACGCAAUUU
eGFP	ACUUCAAGGAGGACGGCAAUU
**DNA oligo probes for small RNA northern blot**	
siRNA specific oligos	GGTGCCCGACATGTCTGGAAA<br>CTTCAGCATCCCGTCGGTAGC<br>TTCTGACACCTTTCATCGCCT<br>GTTAAACTCTGAAGGCACCTT<br>AGGTCAATGATGGTCGGCGCC
piRNA specific oligos	AAGCTAGCTTTGCCAGGTCCGCGTTGT<br>ACGTTTCCGGTTCCACAGGTGCAGTTGTA<br>GTGCCAACGTTTCCGGTTCCACAGGTG<br>TCCGCGTTGTCGATGACTCCTTTCACG<br>TGTTCCGTTTCGGCACGTCAAGGCTGCC<br>TATCCTCGAGCATCCGTAGTGTGGCCTC
